# Effectiveness of Personalized Feedback Alone or Combined with Peer Support to Improve Physical Activity in Sedentary Older Malays with Type 2 Diabetes: A Randomized Controlled Trial

**DOI:** 10.3389/fpubh.2015.00178

**Published:** 2015-07-13

**Authors:** Shariff-Ghazali Sazlina, Colette Joy Browning, Shajahan Yasin

**Affiliations:** ^1^Department of Family Medicine, Faculty of Medicine and Health Sciences, Universiti Putra Malaysia, Serdang, Selangor, Malaysia; ^2^Royal District Nursing Service Limited, RDNS Research Institute, St. Kilda, VIC, Australia; ^3^Jeffrey Cheah School of Medicine and Health Sciences, Monash University Malaysia, Bandar Sunway, Selangor, Malaysia

**Keywords:** type 2 diabetes mellitus, physical activity, personalized feedback, peer support, elderly Malays

## Abstract

**Introduction:**

Regular physical activity is an important aspect of self-management among older people with type 2 diabetes but many remain inactive. Interventions to improve physical activity levels have been studied but few studies have evaluated the effects of personalized feedback (PF) or peer support (PS); and there was no study on older people of Asian heritage. Hence, this trial evaluated whether PF only or combined with PS improves physical activity among older Malays with type 2 diabetes (T2DM) compared to usual care only.

**Materials and methods:**

A three-arm randomized controlled trial was conducted in a primary healthcare clinic in Malaysia. Sixty-nine sedentary Malays aged 60 years and older with T2DM who received usual diabetes care were randomized to PF or PS interventions or as controls for 12 weeks with follow-ups at weeks 24 and 36. Intervention groups performed unsupervised walking activity and received written feedback on physical activity. The PS group also received group and telephone contacts from trained peer mentors. The primary outcome was pedometer steps. Secondary outcomes were self-reported physical activity, cardiovascular risk factors, cardiorespiratory fitness, balance, quality of life, and psychosocial wellbeing.

**Results:**

Fifty-two (75.4%) completed the 36-week study. The PS group showed greater daily pedometer readings than the PF and controls (*p* = 0.001). The PS group also had greater improvement in weekly duration (*p* < 0.001) and frequency (*p* < 0.001) of moderate intensity physical activity, scores on the Physical Activity Scale for Elderly (*p* = 0.003), 6-min walk test (*p* < 0.001), and social support from friends (*p* = 0.032) than PF and control groups.

**Conclusion:**

The findings suggest that PF combined with PS in older Malays with T2DM improved their physical activity levels, cardiorespiratory fitness, and support from friends.

**Trial registration:**

Current Controlled Trials ISRCTN71447000.

## Introduction

Type 2 diabetes (T2DM) is a common non-communicable disease (NCD) in older people and is becoming a global health problem (1). In 2010, about 106 million people aged ≥60 years had T2DM worldwide consistent with aging population and increasing obesity (2). The prevalence of T2DM is projected to increase to 200 million older people by 2030. It is associated with significant morbidity, disability, and mortality, and the health expenditure is highest among people aged ≥60 years (3).

Regular physical activity is a cornerstone in the management of T2DM, which improves glucose homeostasis and reduces risk of diabetes-related morbidity and mortality (4, 5). Older people especially with chronic NCDs, such as T2DM, do benefit from regular physical activity (6, 7). Despite the increasing evidence of the health benefits for regular physical activity, many older people with T2DM remain sedentary (8, 9). Sedentary older people with T2DM are at increased risk of cardiovascular and coronary events (10), and diminished physical function (11). So, sedentary lifestyle should be discouraged in older members of the community (7, 12).

Interventions promoting physical activity in sedentary people with T2DM have been widely studied. Feedback on behavior change is frequently used to promote physical activity and most studies used motion-sensor devices (accelerometer or pedometer) (13–17). Findings of these studies varied in their effectiveness in increasing physical activity and reducing glycosylated hemoglobin (HbA1c). Another strategy to change behavior is using peer support (PS) in the management of T2DM (18–21). The key functions of PS identified by the World Health Organization (22) and Peers for Progress (23) included “assistance in applying disease management and prevention plans in daily life, emotional and social support, linkage to clinical care and on-going support” (p. i64) (23). Most studies on PS in T2DM focused on diabetes self-management education and support, and these studies showed improved HbA1c (19) and self-care behavior including physical activity (19–21). However, few studies focused on promoting physical activity in older people with T2DM. The rapid increase in the incidence of T2DM (1) and a shift toward an aging population warrants the need for an intervention program to improve the functional status of older people with T2DM (24).

Older people with T2DM often have low physical activity levels (25). Those who are less active have poorer glycemic control (9). Previous systematic reviews, including our own, found no studies that promoted physical activity among sedentary older Malays with T2DM and no studies that compared feedback in combination with PS (26–28). In Malaysia, the prevalence of T2DM increased from 8.2% in 1996 to 14.9% in 2006, with the highest proportion (26.1%) among people aged 60–64 years (29). Older Malaysians with T2DM had low physical activity levels and were more likely to have poorer glycemic control (9). Therefore, we evaluated the effectiveness of personalized feedback (PF) about physical activity patterns alone or in combination with PS, in addition to usual diabetes care in improving physical activity levels in sedentary older Malays with T2DM. We also evaluated the effectiveness of these interventions on glycosylated hemoglobin, other cardiovascular risk factors, functional status, quality of life, and psychosocial wellbeing.

## Materials and Methods

### Research design and participants

The Monash University Human Research Ethics Committee (CF10/3191 – 2010001702) and Medical Research Ethics Committee, Ministry of Health, Malaysia (NMRR-10-1107-7328) approved this study and the study was conducted in accordance with the principles of the Declaration of Helsinki. This trial was registered with the Malaysian National Medical Research Registry, Ministry of Health, Malaysia (registration number NMRR-10-1107-7328), and with Current Controlled Trial Ltd. (registration number ISRCTN71447000).

This was a three-arm randomized trial conducted over 36 weeks with a 1:1:1 allocation into three groups:

1. PF about physical activity patterns;
2. PF about physical activity patterns combined with PS;
3. Control group, receiving only usual diabetes care (CG).

Patients were recruited from an urban primary care clinic in Selangor, Malaysia between January and April 2012. We recruited Malay patients because they had worse glycemic and metabolic control (30) and the lowest prevalence of recommended adequate exercise compared with other ethnic groups (31). Eligible participants were community-dwelling Malays aged ≥60 years, diagnosed with T2DM for ≥1 year, having a sedentary lifestyle [engaging in physical activity <150 min a week of moderate intensity (7)], and with follow-up care for their T2DM ≥ two visits in the last 12 months. The exclusion criteria are shown in Table 1, and were used to ensure safe participation in physical activity.

**Table 1:** **Exclusion criteria for participant recruitment**.

Had recent adjustment in the treatment regime needing increase dose of medication in the last 2 months
Fasting blood glucose of >13 mmol/L
Presence of cognitive impairment (Elderly Cognitive Assessment Questionnaire ≤7)
Had uncontrolled hypertension (blood pressure ≥180/100 mmHg)
Presence of coronary artery syndrome
Presence of hemiparesis or hemiplegia
Known advanced osteoarthritis or conditions deterring walking activity
Presence of psychiatric disorders (such as depression, anxiety, psychosis)
Has complications of diabetes (such as proliferative retinopathy, renal impairment)
Presence of uncontrolled respiratory conditions (such as asthma or chronic obstructive pulmonary disease)
Known hearing impairment
Known visual impairment (visual acuity worse than 6/18 after optical correction)
Lives in residential homes

The recruitment involved placing notices at the clinic, personal communication with the patients by the clinic staff, and contacting potential participants via telephone. Participants were screened for eligibility and safety to participate and provided written informed consent before baseline assessment. The recruitment was conducted in the same clinic. Participants from different groups attended their scheduled clinic visits on different days to minimize cross contamination. Peer mentors (whom were patients themselves) were also instructed to share the intervention with their allocated peers only. This study was performed as planned in the study protocol published elsewhere (32).

### Interventions

The interventions incorporated constructs of Social Cognitive Theory to promote change in behavior from sedentary behavior to being physically active through social support and self-efficacy (33). PF and PS groups engaged in a 12-week regular unsupervised walking activity. The participants performed gradual walking activity toward the recommended 30 min a day on ≥5 days in a week at moderate intensity and monitored their walking activity intensity using the Talk Test (34, 35).

#### Personalized Feedback About Physical Activity Patterns

Participants in the PF and PS groups received structured PF and usual diabetes care. The feedback comprised participants’ physical activity patterns (based on the calculated minutes spent walking in a week each month) provided in three one-to-one sessions with the first author during monthly clinic visits. Their attending doctors at the clinic provided the usual diabetes care.

#### Peer Support

The participants in the PS group received support from peer mentors in addition to the PF and usual diabetes care. Peer mentors are “individuals who successfully coped with the same condition and can be a positive role model” (p. i26) (36). The peer mentors were volunteers aged ≥60 years with T2DM who lived in the same community as the participants. They motivated and provided support to the participants to walk regularly based on the feedback through three face-to-face and three telephone contacts over the 12 weeks. The protocol for the peer mentors included recruitment, training, and supervision, and has been described elsewhere (32).

#### Control Group

Participants in the control group received usual diabetes care and acted as a comparison group. The usual diabetes care practice in this study was based on the Malaysian guideline on the management of T2DM, which includes education on lifestyle modification (including diet and physical activity), medications, and self-care management (37). During the 12-week intervention, the control group attended the clinic at a monthly interval to refill their prescriptions. All participants in this study were given pedometers to objectively measure physical activity levels, not as a motivating tool. The motivating factor for the intervention groups was to achieve the recommended duration and frequency of the walking activity. The pedometer readings were not assessed during the 12 weeks of intervention.

### Outcomes

#### Primary Outcome

The primary outcome was physical activity level measured using a reliable and valid pedometer (Yamax Digi-Walker^®^ CW 700/701, Japan) (38). Participants were taught the correct use of the pedometer and were instructed to wear it during their waking hours. All participants were instructed to record total daily step counts in a physical activity diary over 7 days at baseline, and at weeks 12, 24, and 36. The total step counts recorded were divided by the 7 days of assessment to estimate the average steps/day. Based on current best practice, the step counts should be estimated using at least 3 days of readings if the participants did not complete the 7 days assessment (39). The pedometer has a 2-week memory recall that allowed the research team to recover participants’ daily step counts over the last week before their three-monthly assessments if no readings were recorded in their diary.

#### Secondary Outcomes

The secondary outcomes included subjective measure of physical activity: weekly duration and frequency of structured physical activity from the diary, and Physical Activity Scale for the Elderly (PASE) (40). The PASE questionnaire included leisure-time, household and work-related activities, and duration of daily activities done while seated representing sedentary behavior.

Other secondary outcomes were cardiovascular risk factors, functional status, quality of life, and psychosocial wellbeing. The cardiovascular risk factors included glycosylated hemoglobin (HbA1c), blood pressure, body composition (weight, body mass index, waist circumference, body fat percentage), and lipid profiles (low-density lipoprotein cholesterol, high-density lipoprotein cholesterol, triglycerides). Functional status (cardiorespiratory fitness and balance) were measured by the 6-min walk test (41) and the timed up and go test (42), respectively. The 6-min walk test requires the participant to walk for 6 min and the distance in meters was recorded. The protocol adhered to the recommendations of the American Thoracic Society Guideline (43). There are no standard cut-off values to interpret the results of the 6-min walk test. However, it is recommended that comparison based on the mean changes in the distance walked be made following an intervention (43, 44). Quality of life was represented by the physical component summary (PCS) and mental component summary (MCS) scores of the SF-12 Health Survey (45). Psychological wellbeing was measured using the General Health Questionnaire-12 (46), and perceived social support from significant other family and friends was measured by the Multidimensional Scale for Perceived Social Support (MSPSS) (47). The Self-Efficacy for Exercise Scale was also included in the measurement suite (48).

All outcomes were measured at four time points: baseline, week-12 post-intervention, and follow-up at weeks 24 and 36.

#### Adverse Events

We assessed for any occurrence of adverse events that might be due to the interventions that included falls, hypoglycemic episodes, life threatening events, and hospitalization. Participants were asked to report such events spontaneously and we collected additional information during the assessment time points.

### Sample size

Sample size was estimated using G*Power version 3.1.3 software (49) at a statistical significance level of 5 and 80% power. In this study, the primary outcome was pedometer-determined physical activity. We calculated a sample size of 17 per group based on the difference in daily step counts from 4,099 ± 2,152 (pre-intervention) to 7,976 ± 4,118 (post-intervention) following an intervention delivered by peer mentors to promote physical activity in adults with T2DM (18). A minimum sample size of 20 per group was required after considering 20% loss to follow-up.

### Randomization

Eligible participants were sequentially numbered and allocated into one of the groups. The randomization schedule was computer generated using random block sizes of three with an allocation ratio of 1:1:1 (50). The principal author conducted the assignment of interventions after baseline assessments were collected. The blinding of the participants was not possible because of the nature of the intervention. The group allocation was concealed from other research team members not involved in the assignment of the intervention or data analysis, but involved in recruitment and data collection. All participants attended the clinic at time of randomization (at baseline) and at three-monthly intervals for 36 weeks between May 2012 and January 2013.

### Statistical analysis

Data were analyzed using IBM Statistical Package for Social Sciences (SPSS) version 20.0 (IBM Corp., Armonk, NY, USA). Participants’s demographic characteristics, clinical history, and baseline variables were described using means and SDs or median and interquartile range, and frequencies and percentages. The participants’ baseline characteristics were compared using Chi-square or Exact tests and one-way ANOVA or Kruskal–Wallis tests. *Post hoc* tests were conducted to determine significant relationships between groups.

Missing data were not imputed and incomplete data analysis was conducted using linear mixed modeling (LMM) employing intention-to-treat principles (51). The effectiveness of the interventions between groups at baseline, weeks 12, 24, and 36 was determined for all outcomes. An exploratory model building strategy using diagonal covariance structure for repeated measures was performed to select the final model for outcomes (52). The three groups and repeated measures (four time points) of the outcomes were included in the model as the fixed effect factors and were estimated using maximum likelihood method as it provides more accurate estimates of fixed regression parameters. There was no random effect in this study because it was conducted in one primary care clinic and the participants were recruited from the same sample of population.

Results from the final model were presented as adjusted mean and SE for each group at the four time points. Contrast tests were performed on outcomes with significant differences between groups over time and were presented as standardized estimates (β), SE, and 95% confidence intervals. Time point at baseline served as the reference. The analysis controlled for covariates that differed between groups at baseline, which were treatment modalities for diabetes, types of prescribed medication, and SF-12 MCS scores. Adjusted *R*-squared was calculated and effect sizes were reported according to Cohen’s definition: *R*^2^ = 0.14 is a small, *R*^2^ = 0.36 is a medium, and *R*^2^ = 0.51 is a large effect size (53). The significant level was set at *p* value <0.05. The social support and self-efficacy measures were potential mediators on the pedometer-determined physical activity; but it was beyond the scope of this study to perform a mediation analysis.

## Results

### Baseline characteristics

**Figure 1:**
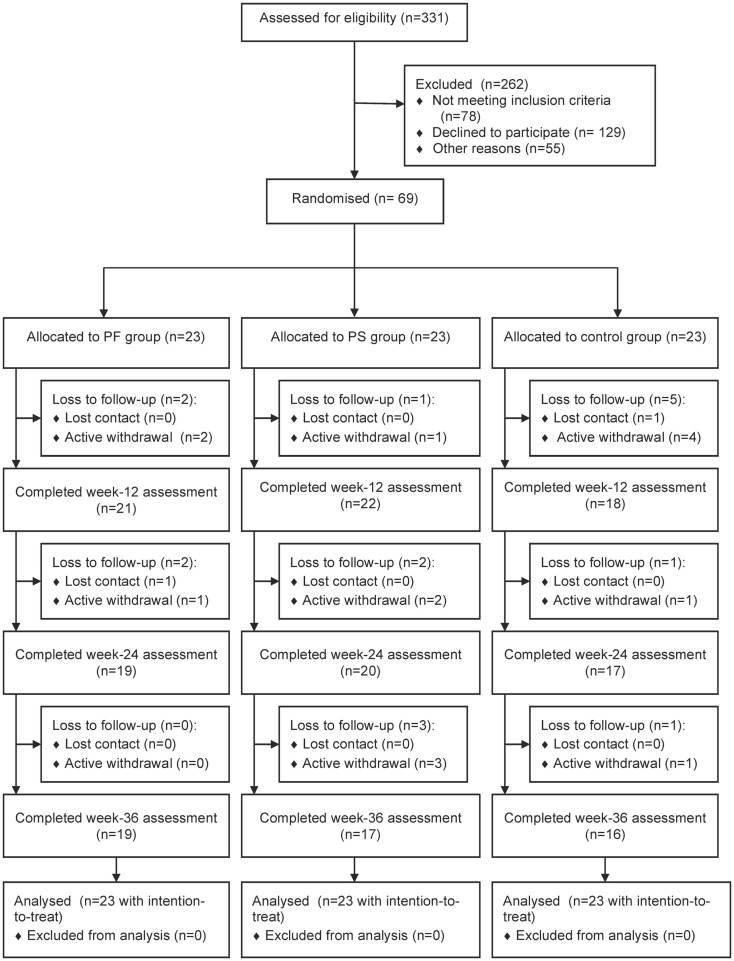
**Flow of study participants**.

Figure 1 shows the flow of study participants. We approached 331 patients and 253 fulfilled the inclusion criteria. Sixty-nine patients enrolled in the study and 23 were randomized to each group. Baseline demographic and clinical characteristics were comparable except for treatment modalities for diabetes, types of prescribed medication, and SF-12 MCS scores. The control group had significantly greater mean types of prescribed medication and a higher proportion was on both oral antihyperglycemic agents and insulin, while the PS group had higher mean SF-12 MCS scores (Table 2). Participants were recruited between January and April 2012 and they attended the clinic visits from May 2012 to January 2013 during this study.

**Table 2:** **Baseline characteristics**.

	Total (*N* = 69)	PF (*n* = 23)	PS (*n* = 23)	Control (*n* = 23)
Age (years)^a^	64.00 (7.00)	63.00 (8.00)	64.00 (7.00)	63.00 (7.00)
Men^b^	37 (53.6)	14 (60.9)	12 (52.2)	11 (47.8)
Married^b^	57 (82.6)	20 (87.0)	18 (78.3)	19 (82.6)
Co-reside with adult children^b^	52 (75.4)	19 (82.6)	14 (60.9)	19 (82.6)
Highest education				
Secondary education^b^	51 (73.9)	16 (69.6)	18 (78.3)	17 (73.9)
Diabetes duration (years)^a^	9.00 (9.50)	10.00 (9.00)	9.00 (11.00)	6.00 (9.00)
Treatment modalities for diabetes^b^				
• Diet alone	1 (1.4)	0	1 (4.3)	0
• Diet and oral AHA(s)	51 (73.9)	14 (60.9)	19 (82.6)	18 (78.3)
• Oral AHA(s) only	3 (4.4)	0	0	3 (13.0)
• Oral AHA(s) and insulin	14 (20.3)	9 (39.1)*	3 (13.0)*	2 (8.7)*
Prescribed medications^c^	4.58 (1.54)	3.78 (1.57)*	4.83 (1.19)*	5.13 (1.55)*
Pedometer readings (steps/day)^c^	3549.77 (468.48)	3771.78 (486.64)	3681.91 (486.34)	3341.78 (486.64)
Weekly duration of moderate intensity physical activity (minutes/week)^a^	0.00 (0.00)	0.00 (0.00)	0.00 (0.00)	0.00 (0.00)
Weekly frequency of moderate intensity physical activity (days/week)^a^	0.00 (0.00)	0.00 (0.00)	0.00 (0.00)	0.00 (0.00)
PASE scores^c^	127.58 (54.28)	142.00 (62.05)	118.87 (56.79)	121.87 (41.25)
PASE daily activities while seated (hours/day)^a^	2.00 (2.00)	2.00 ± 3.00	2.00 ± 2.00	2.00 ± 1.00
HbA_1_c (%)^a^	8.10 (1.90)	8.30 (1.70)	8.10 (2.00)	8.10 (2.70)
Systolic BP (mmHg)^c^	138.30 (13.32)	137.35 (6.42)	138.52 (13.32)	139.04 (10.68)
Diastolic BP (mmHg)^a^	80.00 (10.00)	78.00 (17.00)	80.00 (10.00)	80.00 (10.00)
Weight (kg)^c^	70.19 (12.85)	70.02 (13.10)	69.92 (12.34)	70.62 (13.65)
BMI (kg/m^2^)^a^	26.80 (5.50)	26.60 (4.40)	27.00 (5.50)	26.10 (7.00)
Waist circumference (cm)^a^				
Men	93.00 (7.00)	94.25 (10.00)	91.50 (4.75)	96.00 (9.00)
Women	91.00 (15.25)	90.00 (13.00)	93.00 (16.00)	91.00 (19.00)
Body fat (%)^a^				
Men	24.90 (4.35)	24.80 (5.88)	24.90 (4.98)	26.20 (3.00)
Women	38.75 (1.97)	37.80 (7.25)	40.60 (13.60)	37.30 (9.28)
LDL-C (mmol/L)^c^	3.22 (0.93)	3.11 (0.72)	3.30 (0.94)	3.23 (1.13)
HDL-C (mmol/L)^a^				
Men	1.00 (0.30)	1.15 (0.33)	0.95 (0.18)	1.00 (0.40)
Women	1.10 (0.30)	1.20 (0.20)	1.10 (0.30)	1.15 (0.42)
Triglycerides (mmol/L)^a^	1.60 (0.90)	1.60 (1.00)	1.40 (1.40)	1.60 (1.40)
6-min walk test (meters)^c^	10.00 (2.50)	236.00 (65.85)	216.52 (53.81)	196.52 (68.46)
Timed up and go test (s)^a^	46.29 (15.18)	10.00 (2.00)	9.00 (2.00)	9.00 (3.00)
SF-12 PCS scores^a^	57.93 (10.46)	49.16 (6.91)	44.98 (16.34)	46.74 (17.01)
SF-12 MCS scores^a^	0.00 (1.00)	56.74 (9.98)*	60.10 (10.52)*	56.09 (10.7)*
GHQ-12 scores^a^	0.00 (1.00)	0.00 (1.00)	0.00 (0.00)	0.00 (1.00)
MSPSS scores^a^				
Significant others	6.00 (1.00)	6.00 (1.25)	6.00 (0.75)	6.00 (0.50)
Family	6.00 (0.63)	6.00 (0.75)	6.00 (1.00)	6.00 (0.75)
Friends	5.00 (2.75)	5.00 (1.50)	5.50 (3.00)	5.00 (2.75)
SEES scores^c^	6.64 (1.46)	6.73 (0.98)	6.75 (1.45)	6.44 (1.86)

*PF, personalized feedback only; PS, PF and peer support*.

*Data are ^a^median (IQR) (Kruskal–Wallis), ^b^frequency (%) (Chi-square/Exact test), or ^c^mean (SD) (one-way ANOVA)*.

AHA, anti hyperglycemic agents; PASE, Physical Activity Scale for Elderly; PCS, physical component summary; MCS, mental component summary; GHQ, General Health Questionnaire; MSPSS, Multidimensional Scale for Perceived Social Support; SEES, Self-Efficacy for Exercise Scale; *p value < 0.05 = statistical significance.

Fifty-two (75.4%) participants completed the study. Significantly more women than men were lost to follow-up (*p* = 0.021). Also, those lost to follow-up had higher weight (*p* = 0.018) and BMI (*p* = 0.019), lower cardio respiratory fitness (*p* = 0.001), balance (*p* = 0.021), PASE scores (*p* = 0.003), SF-12 PCS scores (*p* = 0.040), and Self-Efficacy Score for Exercise (*p* = 0.017) than those who completed this study (results not shown). A trend showed that more participants from the control group were lost to follow-up (41.2%) than the PF (23.5%) and PS (35.3%) groups (*p* = 0.579). All participants adhered to wearing the pedometer, but the number of participants who completed the diary declined over time: 59/61 (96.7%) at week 12, 53/56 (94.6%) at weeks 24, and 48/52 (92.3%) at week 36 (data not shown). The weekly duration and frequency of physical activity contributed to the missing data of this study. All participants randomized into this study were included in the analysis using intention-to-treat principles.

### Primary outcome

**Figure 2:**
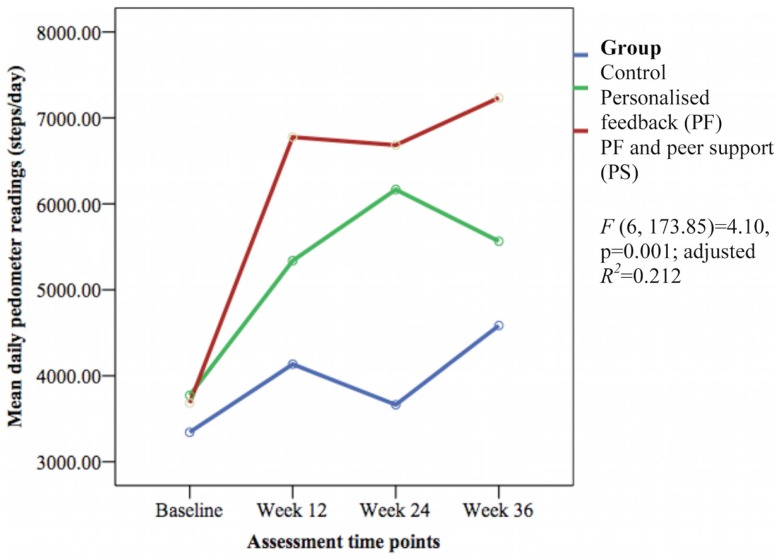
**Mean differences on daily pedometer steps between groups over time**.

The mean daily pedometer readings were significantly different between groups over time [*F*(6, 173.85) = 4.10, *p* = 0.001; adjusted *R*^2^ = 0.212] with a small effect size (Figure 2). The PS group showed significantly greater daily pedometer readings compared to the PF group (week 12: β 1416.12 ± SE 621.62 steps/day, 95% CI 189.19–2643.05, *p* = 0.024; week 36: β 1416.67 ± SE 661.68 steps/day, 95% CI 110.78–2722.57, *p* = 0.034) and the control group (week 12: β 2265.85 ± SE 642.93 steps/day, 95% CI 997.05–3534.66, *p* = 0.001; week 24: β 2586.31 ± SE 660.33 steps/day, 95% CI 1283.21–3889.41, *p* < 0.001; and week 36: β 2084.94 ± SE 685.25 steps/day, 95% CI 732.69–3437.18, *p* = 0.003).

### Secondary outcomes

**Figure 3:**
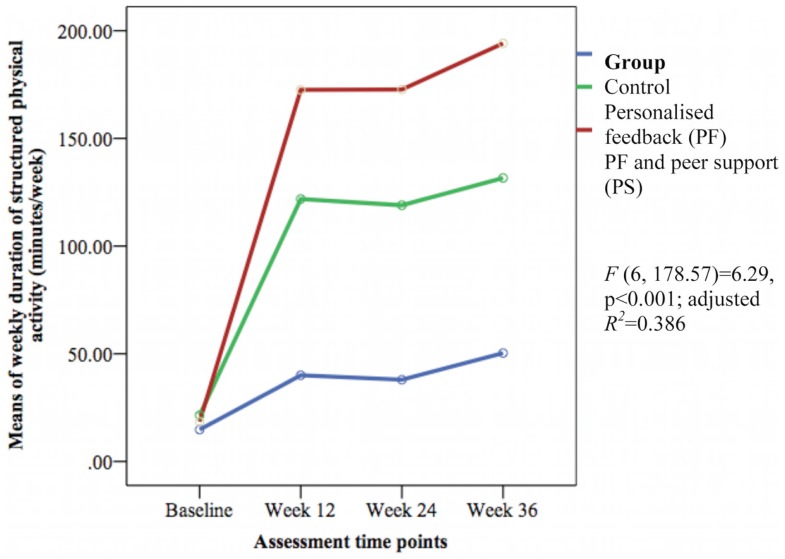
**Mean differences on weekly duration of structured physical activity between groups over time**.

**Figure 4:**
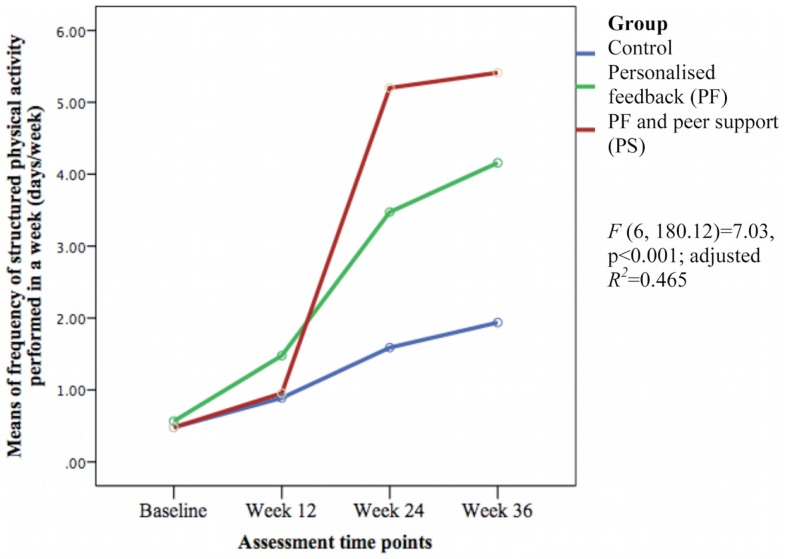
**Means differences on weekly frequency of structured physical activity between groups over time**.

**Figure 5:**
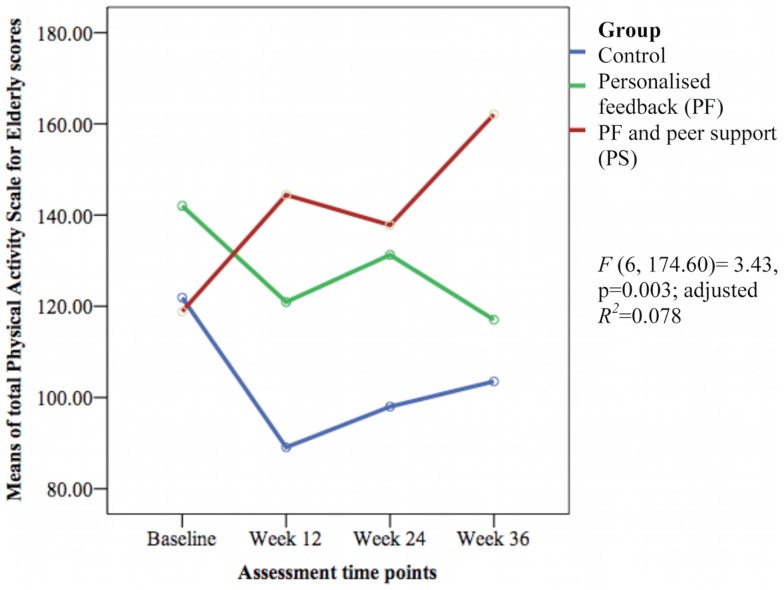
**Mean differences on Physical Activity Scale for Elderly (PASE) scores between groups over time**.

The groups differed over time on weekly duration [*F*(6, 178.57) = 6.29, *p* < 0.001; adjusted *R*^2^ = 0.386] (see Figure 3), frequency of structured physical activity [*F*(6, 180.12) = 7.03, *p* < 0.001; adjusted *R*^2^ = 0.465] (see Figure 4), and PASE scores [*F*(6, 174.60) = 3.43, *p* = 0.003; adjusted *R*^2^ = 0.078] (see Figure 5) but not on PASE duration of daily activities while seated (*p* = 0.629). The PF and PS groups had greater weekly duration of structured physical activity than the control group at post-intervention and at follow-up. The PS group also had greater weekly duration of structured physical activity than the PF and control groups during this study.

The weekly frequency of physical activity was not significantly different between the groups at post-intervention. The PF and PS groups showed greater increases in the weekly frequency of structured physical activity than the control group during follow-up. The PS group had greater increase than the PF group and control group at week 24 but this was not sustained at week 36. The PASE scores were greater in the PS group than in the control group during this study. The PS group had greater scores than the PF and control groups at week 12 and 36.

**Figure 6:**
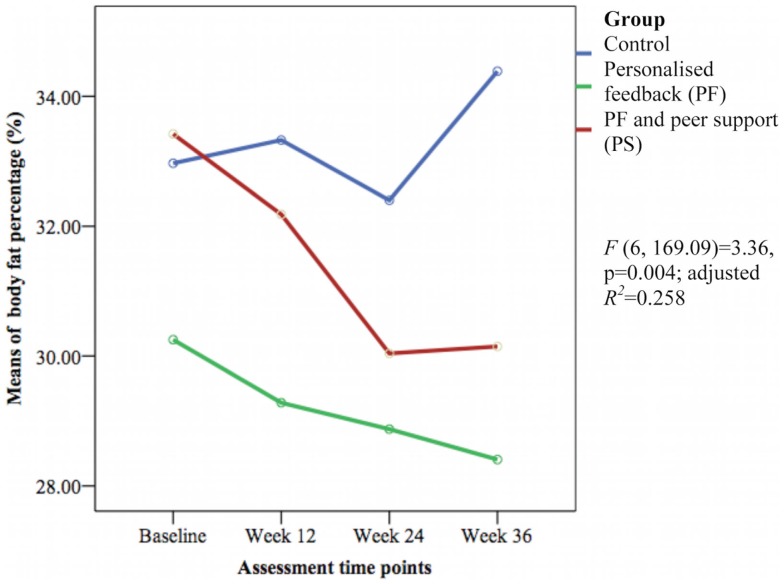
**Mean differences on body fat percentage between groups over time**.

The HbA1c level and other cardiovascular disease risk factors showed no group differences over time except for the mean body fat percentage [*F*(6, 169.09) = 3.36, *p* = 0.004; adjusted *R*^2^ = 0.258] (see Figure 6). The PS group had greater reductions in body fat percentage compared with the PF and control groups during this study. The PF and PS groups also had greater reductions at week 12 and 36 than the control group.

**Figure 7:**
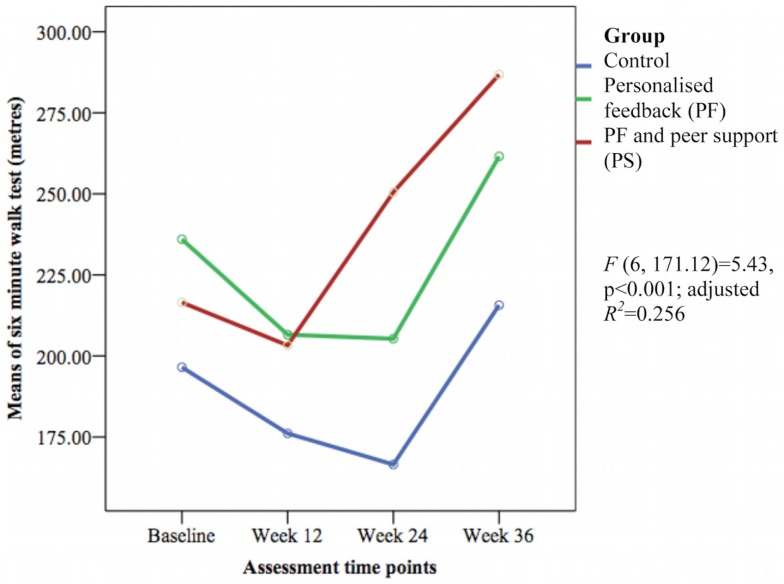
**Mean differences on distance walked in 6 min between groups over time**.

**Figure 8:**
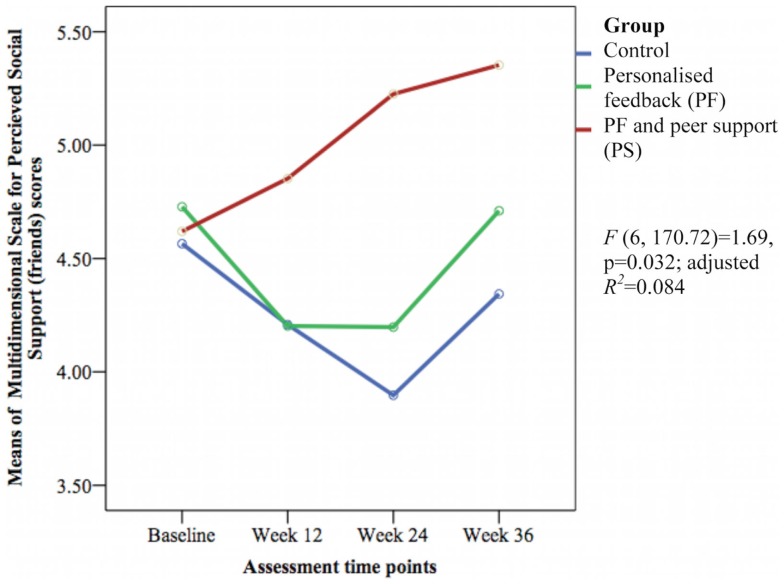
**Mean difference of Multidimensional Scale for Perceived Social Support (friends) scores between groups over time**.

Only the 6-min walk test representing cardiorespiratory fitness [*F*(6, 171.12) = 5.43, *p* < 0.001; adjusted *R*^2^ = 0.256] (see Figure 7) and perceived social support from friends [*F*(6, 170.72) = 1.69, *p* = 0.032; adjusted *R*^2^ = 0.084] (see Figure 8) showed significant differences between groups over time. The PS group showed greater increase in cardiorespiratory fitness and social support from friends at weeks 24 and 36 than the control group, but not at week 12. The PS group also showed greater increase in cardiorespiratory fitness at week 24 than the PF group.

### Adverse events

None of the participants from the present study sustained any injury or experienced any adverse events due to the intervention.

## Discussion

The current study revealed that a PF combined with PS intervention significantly improved physical activity level (pedometer readings, weekly duration of structured physical activity, and PASE scores) in sedentary older Malays with T2DM at post-intervention. These were sustained during this study when compared with the PF only and control groups. The PS group significantly improved weekly frequency of structured physical activity, cardiorespiratory fitness, and social support from friends during the follow-up but not at post-intervention when compared with the PF and control group. Both the PS and PF groups significantly improved pedometer readings, weekly duration of structured physical activity, and body fat percentage at post-intervention and follow-up when compared to the control group. However, the PF and PS groups did not improve HbA1c and other secondary outcomes.

We found that PS improved the physical activity level among our older participants with T2DM, which is consistent with previous studies (18, 19, 21). These studies showed significant increases in pedometer steps/day (18), minutes/week (21), and days/week (19) of structured physical activity. The Canadian quasi-experimental First Step Program promoting physical activity using self-efficacy, social support, and feedback over 16 weeks found an intervention delivered by PS was equally effective in increasing daily pedometer readings as those delivered by healthcare professionals (18). However, no comparison with controls was made and sustainability of the intervention was not evaluated. Both U.S.-based and Chinese-based randomized controlled trials (RCTs) that included peer mentors to promote physical activity as part of a multiple health behavior program showed greater minutes/week (21) and days/week (19) of structured physical activity when compared to controls. However, these interventions were focused on diabetes self-management education and support, and they measured physical activity using a questionnaire (19, 21).

In our study, we found that at week 12, there was a substantial improvement in pedometer readings and duration of structured physical activity in the PS and PF groups. However, the mean frequency of physical activity was lower at week 12. Based on the descriptive data that we analyzed (data not shown) at week 12, participants in both PF and PS groups performed between 0 and 7 times a week of moderate intensity physical activity. They engaged in longer duration of physical activity, i.e., longer than 30 min of each walking exercise, but performed less frequently in a week, which is reflected by the average of longer duration and lower frequency of physical activity found in this study. The recommendation for older people is to perform regular physical activity of at least 30 min on 5 days each week (7), which the participants did not achieve at this time point. It is unclear why the participants did not achieve compliance with recommendations at week 12. The differences between groups at week 12 were also not significant.

The PASE scores in our study were significantly greater in the PS group than in the PF and control groups. Similarly, previous RCTs with PS interventions reported greater physical activity levels based on self-reported physical activity scales compared with controls (19–21). Unfortunately, the scales used in these studies varied making comparison difficult. We did not find differences between groups in the sedentary behavior. None of the previous studies among people with T2DM with PS programs measured sedentary behavior.

Peer support increased cardiorespiratory fitness and social support from friends during the follow-up period in the present study. Comparison with previous studies could not be done because no studies on feedback or PS measured cardiorespiratory fitness or social support in older people with T2DM following physical activity interventions. A meta-analysis found regular structured physical activity improved cardiorespiratory fitness in adults with T2DM (54). In contrast to our study, a quasi-experimental study of Chinese women that evaluated the effect of group-based Tai Chi exercise on social support reported significant increased MSPSS (family) and MSPSS (significant others) scores but not MSPSS (friends) score (55). Our process evaluation showed that participants in the PS group considered their newly acquainted peers as their new friends, which may account for the increase in MSPSS (friends) scores.

In our study, the interventions did not improve HbA1c and other CVD risk factors. Previous studies using a blood glucose self-monitoring chart as feedback (17) and PS (19) to promote physical activity in adults with T2DM showed significant reduction in HbA1c as opposed to our findings. However, the improvements of HbA1c in these studies were short-term. We found decreased body fat percentage in the PF and PS groups. Comparison with previous studies that incorporated feedback or PS among adults with T2DM could not be made as body fat percentage was not an outcome in those studies.

The PS group showed improved physical activity levels in the short-term, which was sustained when compared to the PF or control groups in our study. Peers have been shown to influence one another, which results in adopting similar behaviors (56). Our process evaluation found that the participants in the PS group developed their own social network outside the study. The interaction could have contributed to the adherence to regular physical activity. This “flow on” effect is an important finding from this study where participants were able to take the experience of the intervention and translate it into sustainable activities. The social interaction between peer mentors and participants in the PS group could also influence greater perceived social support from friends scores. Further, friends have been shown to correlate positively with older people’s participation in leisure activities (57).

Sedentary behavior was evaluated to provide a comprehensive definition of physical activity level in the present study. We found most participants in all groups reported spending less than 4 h a day on activities while seated and that remained unchanged during the course of the study. We assessed sedentary behavior using a question from the PASE: hours spent in a day on activities done while seated and not lying down. So, the true extent of sedentary behavior in this study may have been underestimated.

All groups in our study showed a similar reduced trend in HbA1c, weight, BMI, and waist circumference at post-intervention, which could explain why the difference between the groups was not detected. These reductions in the intervention groups could be attributed to increased walking activity. But, in the control group, it is unclear why such reductions were observed, as they did not engage in regular physical activity. A “trial or Hawthorne effect” could explain this observation; a phenomenon where a trial may bring about positive effects on the participants’ behaviors and outcomes (58). The participants may alter their behavior to improve themselves as a result of being in a study. The control group may also have improved their dietary control during the intervention, but we were not able to confirm this as dietary intake was not measured.

Our study showed that older Malays can be motivated to change their behavior even at a later age especially with the influence of peers. There are limited healthcare resources in Malaysia to provide on-going diabetes self-management education and support due to lack of a trained workforce and time constraints. Healthcare providers could empower patients to improve their physical activity level and diabetes self-care through PS groups at the primary care setting, which may prove to be a cost-effective approach.

A PS program is feasible among older Malays with T2DM bridging primary health care and community settings in Malaysia. It had positive effects on physical activity behavior and functional status of our older participants with T2DM. Future trials should investigate the effects of such interventions among other ethnic groups in Malaysia and should be conducted in other settings, for example, in collaboration with community organizations to allow better generalizability of such program in Malaysia. Further, a PS program does require resources but the investment may be relatively lower when older people can be empowered and be more self-reliant to self-manage their diabetes. An economic evaluation of the PS approach needs to be included in future trials promoting physical activity in older people and in people with T2DM with longer follow-up period and rigorous cost analysis. We used physical activity level rather than a clinical indicator such as HbA1c level as the motivating factor to promote physical activity. The use of a clinical indicator that closely reflects the participants’ disease control could plausibly further enhance behavior change and improve clinical outcomes in future trials.

### Strength and weaknesses of the study

A strength of our study was a long follow-up period that allows evaluation of the sustainability of interventions promoting walking activity. Also, pedometer measures physical activity level objectively and allows more accurate classification of physical activity (59). As far as we can ascertain, this is the first RCT that evaluated the effectiveness of feedback in combination with PS in older patients with T2DM in the Malay community.

This study also has some weaknesses. First, the response rate was low. The process of recruitment involved several stages with extensive screening before randomization. Potential participants needed to comply with the study for 9 months. These factors could have discouraged some of the participants from participating. In addition, those who finally enrolled were more likely to be those who were highly motivated to change their behavior (60). This may lead to a recruitment bias.

Second, significantly more men, and participants with lower weight and BMI, and better cardiorespiratory fitness and balance completed this study. Analyses to determine the effect of gender on the outcomes were performed (data not shown), but there were no significant differences in the primary and secondary outcomes between the groups over time.

Finally, sedentary behavior was assessed using activities while seated while true sedentary behavior should include activities while lying down. So, self-reported measures for sedentary behavior should evaluate the time spent on each sedentary activities rather than the time spent on these activities in total (61).

## Conclusion

Personalized feedback about patterns of physical activity in combination with PS significantly improved physical activity level, cardiorespiratory fitness, and social support from friends in older Malays with T2DM when compared with those experiencing usual diabetes care. PF combined with PS approaches should be considered as a viable intervention for older people with T2DM to increase physical activity levels.

## Author Contributions

S-GS, CB, and SY were responsible for the conception and design of the study. S-GS oversaw the implementation of study, compiled the data, and led the data analysis and interpretation. S-GS prepared the first draft of the manuscript. All authors contributed to the subsequent drafts and approved the final version of the manuscript.

## Conflict of Interest Statement

The authors declare that this trial was conducted in the absence of any commercial or financial relationship that can construed as a potential conflict of interest.

## References

[1] International Diabetes Federation. Diabetes Atlas. 4th ed Brussels: International Diabetes Federation (2010).

[2] Shaw JE Sicree RA Zimmet PZ. Global estimates of the prevalence of diabetes for 2010 and 2030. Diabetes Res Clin Pract (2010) 87:4–14. 10.1016/j.diabres.2009.10.007 19896746

[3] Zhang P Zhang X Brown J Vistisen D Sicree R Shaw J Global healthcare expenditure on diabetes for 2010 and 2030. Diabetes Res Clin Pract (2010) 87:293–301. 10.1016/j.diabres.2010.01.026 20171754

[4] Sigal RJ Kenny GP Boulé NG Wells GA Prud’homme D Fortier M Effects of aerobic training, resistance training, or both on glycemic control in type 2 diabetes. Ann Intern Med (2007) 147:357–69. 10.7326/0003-4819-147-6-200709180-00005 17876019

[5] Church TS LaMonte MJ Barlow CE Blair SN. Cardiorespiratory fitness and body mass index as predictors of cardiovascular disease mortality among men with diabetes. Arch Intern Med (2005) 165:2114–20. 10.1001/archinte.165.18.2114 16217001

[6] Chodzko-Zajko WJ Proctor DN Fiatarone Singh MA Minson CT Nigg CR Salem GJ Exercise and physical activity for older adults. Med Sci Sports Exerc (2009) 41:1510–30. 10.1249/MSS.0b013e3181a0c95c 19516148

[7] Nelson ME Rejeski WJ Blair SN Duncan PW Judge JO King AC Physical activity and public health in older adults. Recommendation from the American College of Sports Medicine and the American Heart Association. Circulation (2007) 116:1094–105. 10.1161/CIRCULATIONAHA.107.185650 17671236

[8] Nelson KM Reiber G Boyko EJ. Diet and exercise among adults with type 2 diabetes: findings from the third national health and nutrition examination survey (NHANES III). Diabetes Care (2002) 25:1722–8. 10.2337/diacare.25.10.1722 12351468

[9] Shazwani MNN Suzana S Lim CJ Teh CS Fauzee MZM Lim HC Assessment of physical activity level among individuals with type 2 diabetes mellitus at Cheras Health Clinic, Kuala Lumpur. Malays J Nutr (2010) 16:101–12. 22691857

[10] Yang Y Goh S-Y Tan SB Ho HJ Emmanuel S Wang P The burden of diabetes mellitus in elderly patients from an Asian tertiary hospital. Eur J Intern Med (2012) 23:e1–4. 10.1016/j.ejim.2011.10.017 22153540

[11] Seguin R LaMonte M Tinker L Liu J Woods N Michael YL Sedentary behavior and physical function decline in older women: findings from the women’s health initiative. J Aging Res (2012) 2012:271589. 10.1155/2012/271589 22675631 PMC3364591

[12] Hordern MD Dunstan DW Prins JB Baker MK Singh MAF Coombes JS. Exercise prescription for patients with type 2 diabetes and pre-diabetes: a position statement from exercise and sport science Australia. J Sci Med Sport (2012) 15:25–31. 10.1016/j.jsams.2011.04.005 21621458

[13] Diedrich A Munroe DJ Romano M Promoting physical activity for persons with diabetes. Diabetes Educ (2010) 36:132–40. 10.1177/0145721709352382 20019197

[14] Bjørgaas MR Vik JT Stølen T Lydersen S Grill V. Regular use of pedometer does not enhance beneficial outcomes in a physical activity intervention study in type 2 diabetes mellitus. Metabolism (2008) 57:605–11. 10.1016/j.metabol.2007.12.002 18442621

[15] Furber S Monger C Franco L Mayne D Jones LA Laws R The effectiveness of a brief intervention using a pedometer and step-recording diary in promoting physical activity in people diagnosed with type 2 diabetes or impaired glucose tolerance. Health Promot J Austr (2008) 19:189–95. 19053935 10.1071/he08189

[16] Engel L Lindner H. Impact of using a pedometer on time spent walking in older adults with type 2 diabetes. Diabetes Educ (2006) 32:98–107. 10.1177/0145721705284373 16439498

[17] Allen NA Fain JA Braun B Chipkin SR. Continuous glucose monitoring counseling improves physical activity behaviors of individuals with type 2 diabetes: a randomized clinical trial. Diabetes Res Clin Pract (2008) 80:371–9. 10.1016/j.diabres.2008.01.006 18304674 PMC2430041

[18] Tudor-Locke C Lauzon N Myers AM Bell RC Chan CB McCargar L Effectiveness of the first step program delivered by professionals versus peers. J Phys Act Health (2009) 6:456–62. 19842459 10.1123/jpah.6.4.456

[19] Toobert DJ Strycker LA Barrera M Jr Osuna D King DK Glasgow RE. Outcomes from a multipler risk factor diabetes self-management trial for Latinas: ¡Viva Bien! Ann Behav Med (2011) 41:310–23. 10.1007/s12160-010-9256-7 21213091 PMC3108326

[20] Toobert DJ Glasgow RE Strycker LA Barrera M Jr Ritzwoller DP Weidner G. Long-term effects of the mediterranean lifestyle program: a randomized clinical trial for postmenopausal women with type 2 diabetes. Int J Behav Nutr Phys Act (2007) 4:1. 10.1186/1479-5868-4-1 17229325 PMC1783667

[21] Liu S Bi A Fu D Fu H Luo W Ma X Effectiveness of using group visit model to support diabetes patient self-management in rural communities of Shanghai: a randomized controlled trial. BMC Public Health (2012) 12:1043. 10.1186/1471-2458-12-1043 23198694 PMC3533983

[22] World Health Organization. Peer Support Programmes in Diabetes: Report of a WHO Consultation. Geneva: World Health Organization (2008). Report No.: WHO/CPM/08/01.

[23] Boothroyd RI Fisher EB. Peers for progress: promoting peer support for health around the world. Fam Pract (2010) 27:i62–8. 10.1093/fampra/cmq017 20483801

[24] Hendra TJ Sinclair AJ Improving the care of elderly diabetic patients: the final report of the St Vincent joint task force for diabetes. Age Ageing (1997) 26:3–6. 10.1093/ageing/26.1.3 9143430

[25] Zhao G Ford ES Li C Balluz LS. Physical activity in U.S. older adults with diabetes mellitus: prevalence and correlates of meeting physical activity recommendations. J Am Geriatr Soc (2011) 59:132–7. 10.1111/j.1532-5415.2010.03236.x 21226683

[26] Van den Berg MH Schoones JW Vliet Vlieland TP. Internet-based physical activity interventions: a systematic review of the literature. J Med Internet Res (2007) 9:e26. 10.2196/jmir.9.3.e26 17942388 PMC2047289

[27] Shariff-Ghazali S Browning CJ Yasin S. Interventions to promote physical activity in older people with type 2 diabetes mellitus: a systematic review. Front Public Health (2013) 1:71. 10.3389/fpubh.2013.00071 24392445 PMC3870318

[28] Thomas D Elliott EJ Naughton GA. Exercise for type 2 diabetes mellitus. Cochrane Database Syst Rev (2006) 3:CD002968. 10.1002/14651858.CD002968.pub2 16855995 PMC8989410

[29] Institute for Public Health. The Third National Health and Morbidity Survey (NMHS III) 2006, Diabetes. Malaysia: Ministry of Health, Malaysia (2008).

[30] Lee PY Cheong AT Zaiton A Mastura I Chew BH Sazlina SG Does ethnicity contribute to the control of cardiovascular risk factors among patients with type 2 diabetes? Asia Pac J Public Health (2013) 25:316–25. 10.1177/1010539511430521 22186400

[31] Poh BK Safiah MY Tahir A Siti Haslinda MD Siti Norazlin N Norimah AK Physical activity pattern and energy expenditure of Malaysian adults: findings from the Malaysian adult nutrition survey (MANS). Malays J Nutr (2010) 16:13–37. 22691851

[32] Sazlina SG Browning CJ Yasin S. Promoting physical activity in sedentary elderly Malays with type 2 diabetes: a protocol for randomised controlled trial. BMJ Open (2012) 2:e002119. 10.1136/bmjopen-2012-002119 23161092 PMC3533096

[33] Bandura A Social Foundation of Thought and Action: A Social Cognitive Theory. New Jersey: Prentice-Hall, Inc (1986).

[34] Brawner CC Keteyian SJ Czaplicki TE A method of guiding exercise intensity: the talk test. Med Sci Sports Exerc (1995) 27:S241 10.1249/00005768-199505001-01354

[35] Persinger R Foster C Gibson M Fater DCW Porcari JP. Consistency of the talk test for exercise prescription. Med Sci Sports Exerc (2004) 36:1632–6. 15354048

[36] Heisler M. Different models to mobilize peer support to improve diabetes self-management and clinical outcomes: evidence, logistics, evaluation considerations and needs for future research. Fam Pract (2010) 27:i23–32. 10.1093/fampra/cmp003 19293400 PMC2902359

[37] Ministry of Health. Management of Type 2 Diabetes. 4th ed Malaysia: Ministry of Health, Malaysia (2009).

[38] Strycker L Duncan S Chaumeton N Duncan T Toobert D. Reliability of pedometer data in samples of youth and older women. Int J Behav Nutr Phys Act (2007) 4:4. 10.1186/1479-5868-4-4 17306031 PMC1810544

[39] Tudor-Locke CE Bell RC Myers AM Harris SB Lauzon N Rodger NW. Pedometer-determined ambulatory activity in individuals with type 2 diabetes. Diabetes Res Clin Pract (2002) 55:191–9. 10.1016/S0168-8227(01)00317-5 11850095

[40] Washburn RA Smith KW Jette AM Janney CA. The physical activity scale for the elderly (PASE): development and evaluation. J Clin Epidemiol (1993) 46:153–62. 10.1016/0895-4356(93)90053-4 8437031

[41] Rikli R Jones C The reliability and validity of a 6 minute walk test as a measure of physical endurance in older adults. J Aging Phys Act (1998) 6:363–75.

[42] Podsiadlo D Richardson S The timed “up & go”: a test of basic functional mobility for frail elderly persons. J Am Geriatr Soc (1991) 39:142–8. 10.1111/j.1532-5415.1991.tb01616.x 1991946

[43] American Thoracic Society. ATS statement: guidelines for the six-minute walk test. Am J Respir Crit Care Med (2002) 166:111–7. 10.1164/ajrccm.166.1.at1102 12091180

[44] Enright PL McBurnie MA Bittner V Tracy RP McNamara R Arnold A The 6-min walk test: a quick measure of functional status in elderly adults. Chest (2003) 123:387–98. 10.1378/chest.123.2.387 12576356

[45] Ware J Kosinski M Keller SD. A 12-item short-form health survey: construction of scales and preliminary tests of reliability and validity. Med Care (1996) 34:220–33. 10.1097/00005650-199603000-00003 8628042

[46] Goldberg DP Gater R Sartorius N Ustun TB Piccinelli M Gureje O The validity of two versions of the GHQ in the WHO study of mental illness in general health care. Psychol Med (1997) 27:191–7. 10.1017/S0033291796004242 9122299

[47] Zimet GD Dahlem NW Zimet SG Farley GK The multidimensional scale of perceived social support. J Pers Assess (1988) 52:30–41. 10.1207/s15327752jpa5201_2 2280326

[48] Resnick B Luisi D Vogel A Junaleepa P. Reliability and validity of the self-efficacy for exercise and outcome expectations for exercise scales with minority older adults. J Nurs Meas (2004) 12:235–47. 10.1891/jnum.12.3.235 16138727

[49] Faul F Erdfelder E Lang A-G Buchner A. G*Power 3: a flexible statistical power analysis program for the social, behavioral, and biomedical sciences. Behav Res Methods (2007) 39:175–91. 10.3758/BF03193146 17695343

[50] Dallal GE Block Randomization Generator [Internet] (2012). Available from: http://www.randomization.com/

[51] Newell DJ. Intention-to-treat analysis: implications for quantitative and qualitative research. Int J Epidemiol (1992) 21:837–41. 10.1093/ije/21.5.837 1468842

[52] Hox JJ Multilevel Analysis: Techniques and Applications. Mahwah, NJ: Lawrence Erlbaum Associates (2002).

[53] Leech NL Barrett KC Morgan GA Leech NL IBM SPSS for Intermediate Statistics: Use and Interpretation. New York, NY: Routledge (2011).

[54] Boulé N Kenny G Haddad E Wells G Sigal R. Meta-analysis of the effect of structured exercise training on cardiorespiratory fitness in Type 2 diabetes mellitus. Diabetologia (2003) 46:1071–81. 10.1007/s00125-003-1160-2 12856082

[55] Taylor-Piliae RE Haskell WL Waters CM Froelicher ES. Change in perceived psychosocial status following a 12-week Tai Chi exercise programme. J Adv Nurs (2006) 54:313–29. 10.1111/j.1365-2648.2006.03809.x 16629916

[56] Christakis NA Fowler JH. The collective dynamics of smoking in a large social network. N Engl J Med (2008) 358:2249–58. 10.1056/NEJMsa0706154 18499567 PMC2822344

[57] Toepoel V. Ageing, leisure, and social connectedness: how could leisure help reduce social isolation of older people? Soc Indic Res (2012) 113:355–72. 10.1007/s11205-012-0097-6 23874058 PMC3696179

[58] Braunholtz DA Edwards SJL Lilford RJ. Are randomized clinical trials good for us (in the short term)? Evidence for a “trial effect.” J Clin Epidemiol (2001) 54:217–24. 10.1016/S0895-4356(00)00305-X 11223318

[59] Ewald B McEvoy M Attia J. Pedometer counts superior to physical activity scale for identifying health markers in older adults. Br J Sports Med (2010) 44:756–61. 10.1136/bjsm.2008.048827 18625631

[60] Martinson BC Crain AL Sherwood NE Hayes M Pronk NP O’Connor PJ. Population reach and recruitment bias in a maintenance RCT in physically active older adults. J Phys Act Health (2010) 7:127–35. 20231764 10.1123/jpah.7.1.127 PMC2886498

[61] Healy GN Clark BK Winkler EAH Gardiner PA Brown WJ Matthews CE. Measurement of adults’ sedentary time in population-based studies. Am J Prev Med (2011) 41:216–27. 10.1016/j.amepre.2011.05.005 21767730 PMC3179387

